# Can sequential aerosol technique be used against riverine tsetse?

**DOI:** 10.1371/journal.pntd.0006768

**Published:** 2018-10-11

**Authors:** Reginald De Deken, Jérémy Bouyer

**Affiliations:** 1 Institute of Tropical Medicine, Antwerp, Belgium; 2 Insect Pest Control Laboratory, Joint FAO/IAEA Division of Nuclear Techniques in Food and Agriculture, IAEA, Vienna, Austria; 3 Unité Mixte de Recherche ASTRE ‘Animal, Santé, Territoires, Risques et Ecosystèmes’, Campus international de Baillarguet, Centre de Coopération Internationale en Recherche Agronomique pour le Développement (CIRAD), Montpellier, France; 4 UMR ASTRE, Univ Montpellier, CIRAD, INRA, Montpellier, France; 5 UMR IRD-CIRAD Intertryp, Montpellier, France; Yale School of Public Health, UNITED STATES

## Aim of the paper

For the implementation of the sequential aerosol technique (SAT), several technical constraints such as the calibration of the spray, as well as the meteorological conditions during spray operations, need to be taken into account [1, 2]. Because some of these constraints differ depending on whether savannah or riverine tsetse flies are the target of control, the technique, which has been proven successful in southern Africa against *Glossina morsitans*, cannot be transferred indiscriminately to combat riverine tsetse flies [3]. This paper aims at addressing these elements and identifying which studies are required before using SAT against riverine tsetse species.

## Tuning control operations with the natural reproduction cycle of tsetse flies

Tsetse flies have an unusual reproduction cycle involving the retention of a single egg that develops to the third larval stage within the female fly before being deposited. This larva then quickly pupates and remains underground until it emerges as an adult. The duration of the pupal stage and the time between female emergence and the deposition of the first offspring are predictable given that the ambient temperature is known [2]. The SAT is based on repeated episodes of insecticide spraying at low concentrations, timed at the moments when those females, which have emerged after the latest treatment, are ready to deposit their first pupae. The spraying cycles are continued until the last flies emerging from pupae, which have been deposited prior to spraying episodes, have been sprayed twice.

In the case where several tsetse species are present, spray periodicity is calculated for the species with the shortest interlarval period and treatment length according to the species with the longest pupal period (which are mainly flies from the fusca group). Although sufficient data about the temperature dependence of the development for most tsetse flies from the morsitans and palpalis group are available in the literature, this is not the case for flies of the fusca group occurring in riverine habitats. Fortunately, these flies have little importance as disease vectors. In order to predict the time of the next insecticide application and the likely time of completion of the operation, it may be useful to combine the expected duration of daily larval and pupal development throughout the SAT operation [3,4]. However, this should be done by monitoring temperatures within the gallery forest, which can be as much as 5°C lower than those of the surrounding savannah [5].

## Adapting SAT to the challenging habitats of riverine tsetse species

For adequate dispersal and effective delivery of the aerosol throughout the tsetse habitat, the aircraft needs to fly as low as reasonably safe, requiring the terrain to be fairly flat. Aerial spraying has to be performed during periods of temperature inversion, which tends to occur after sunset on clear nights with light winds when the ground and the air close above the ground become cooler by conduction. This results in a downward transfer of heat energy, and consequently, aerosol droplets are carried down towards the ground. Circulating wind will further disperse the droplets. A complete lack of wind, as well as strong winds, should be avoided. The presence of clouds is also unfavourable because they tend to keep the ground warmer.

In a riverine habitat, favourable conditions are likely to occur more often during the cold dry season due to its relatively stable meteorological conditions and reduced leaf coverage for the following reasons. Water retains heat much longer than soil, and a humid environment is lighter than a dry one. Consequently, temperature inversions will occur less frequently in a humid, tropical environment compared to a subtropical or temperate region and will be seen more rarely in a gallery forest than over open grassland or wooded savannah. This also means that its occurrence must be verified at the control site rather than on the tarmac of the airfield.

Dense vegetation may act as a filter for the aerosol, especially for the more voluminous droplets, leading to a reduction (up to 50%) in the amount of effective pesticide available for impact on the vector [6]. Moreover, reduced wind speed in a tree canopy would reduce impact efficiency and may be a contributing factor for reduced fly mortality in a vegetated environment.

## Technical quality of aerial spraying and its monitoring

The quality of the operation is mainly determined by the density and median volume of the insecticide droplets, spraying drift, and aircraft navigation [7]. The optimal droplet size to control adult tsetse flies is around 15 to 20 μm [8], therefore the insecticide formulation has to include a volatile solvent carrier to reduce droplet size by the evaporation of the solvent. This is particularly important in riverine habitats where droplets greater than 50 μm will have difficulties passing through dense vegetation [6].

The perimeters of spraying blocks are particularly vulnerable to under-dosing, especially in the case of long narrow blocks. This may constitute a problem when spraying gallery forests. Spraying should be executed using global positioning system (GPS)–controlled navigation, whereby altitude, weather conditions, and especially wind speed and direction must be considered [9]. Monitoring of the aerial spraying by assessment of droplet density and diameter on the ground [1,10] is important in challenging sites such as gallery forests.

Entomological surveys and aging of captured female flies are also essential to plan, adjust, and evaluate the results of aerial spraying. Any females captured after the start of the spraying activities that are not nulliparous must have emerged before the previous treatment and thus must have survived the spraying or reinvaded the spraying area [10].

## Insecticidal dosage considering physiological resistant stages and tsetse species susceptibility

The pyrethroid deltamethrin (0.35%) at 0.2 to 0.5 g ai/ha is the only insecticide used in recent years to control tsetse by SAT. SAT uses this residual insecticide at such a low dose that breakdown of the chemical occurs rapidly, enabling tsetse from neighbouring untreated areas to move to the treated area within a couple of days after spraying, remaining unharmed. Studies have shown that the amount of insecticide found on a treated tsetse fly can vary considerably during an SAT operation [10]. Ideally, a tsetse should be killed if exposed to a single droplet of the deltamethrin aerosol. A single droplet with an original volume of 50 μL contains 0.13 ng of deltamethrin, a dose sufficient to kill most *G*. *morsitans* flies [11]. However, the susceptibility to insecticides differs greatly among tsetse species: riverine *G*. *palpalis* spp. would need at least two to four times this dose [11].

Furthermore, female flies of the more resistant species carrying a third stadium larva may tolerate doses of deltamethrin seven times higher than the dose required to kill teneral flies [11] as the lipophilic insecticide is diverted to the nutritive liquid produced by the tsetse’s milk glands. Therefore, when lipophilic insecticides are used, it may be wise to reduce the period between sprays in order to prevent larval development in the female beyond the first instar, although this may require an extra application to cover the whole pupal period.

Although toxicity increases with temperature for most insecticides, the toxicity of deltamethrin contrarily decreases strongly with temperature [12]: e.g., the lethal dose of deltamethrin for 50% of a *G*. *m*. *morsitans* population is 0.03 ng at 18°C and 0.18 ng at 30°C. This characteristic may reduce the efficacy of SAT in tropical compared to subtropical areas.

Environmental monitoring studies [13,14] have shown that the impact of deltamethrin dosed at 0.25 to 0.3 g/ha on local nontarget organisms was generally low. However, especially among aquatic insects and crustaceans, some species have remained at reduced levels, while other species may have even been lost permanently due to the spraying activities. Therefore, any major SAT operation performed in a new environment, either with higher doses of deltamethrin or with any other pesticide, should be accompanied by extensive environmental studies.

In conclusion, it must be emphasized that the sequential aerosol spraying technique needs to be adapted to riparian flies. Before SAT is used to combat riverine tsetse flies, it will be necessary to address the following issues:

- Since the typical habitat of riverine tsetse species is not very favourable for temperature inversion to happen, its occurrence in this habitat must be studied more thoroughly.
- Improve penetration of insecticide formulations in a forested environment through incorporation of more suitable volatile solvents;
- Increase knowledge on temperature-dependent development for some tsetse fly species;
- Develop methods to address possible under-dosing along the perimeters of small spraying blocks;
- Include environmental monitoring to study the impact on local nontarget organisms when increased insecticide dose or higher application frequency is used to control riverine tsetse flies.
